# The Use of Natural Language Processing to Interpret Unstructured Patient Feedback on Health Services: Scoping Review

**DOI:** 10.2196/72853

**Published:** 2025-08-14

**Authors:** Ali Feizollah, Chiu-Yi Lin, Lucy O'Malley, Wendy Thompson, Stefan Listl, Matthew Byrne

**Affiliations:** 1 Division of Dentistry, School of Medical Sciences, Faculty of Biology, Medicine and Health, The University of Manchester, Oxford Road, Manchester, M15 6FH, United Kingdom, 44 1612756783; 2 Section for Oral Health, Heidelberg Institute of Global Health, Heidelberg University Hospital (UKHD), Heidelberg, Germany

**Keywords:** natural language processing, patient-reported outcome measures, patient satisfaction, data mining, quality of health care, social media, machine learning, quality improvement, medical informatics, patient-generated health data

## Abstract

**Background:**

Unstructured patient feedback (UPF) allows patients to freely express their experiences without the constraints of predefined questions. The proliferation of online health care rating websites has created a vast source of UPF. Natural language processing (NLP) techniques, particularly sentiment analysis and topic modeling, are increasingly being used to analyze UPF in health care settings; however, the scope and clinical relevance of these technologies are unclear.

**Objective:**

This scoping review investigates how NLP techniques are being used to interpret UPF, with a focus on the health care settings in which this is used, the purposes for using these technologies, and any impacts reported on clinical practice.

**Methods:**

Searches of the MEDLINE, Embase, CINAHL, Cochrane Database of Reviews, and Google Scholar were conducted in February 2024. No date limits were applied. Eligibility criteria included English-language studies that used NLP techniques on UPF that pertained to an identifiable health care setting or providers. Studies were excluded if human actors solely performed coding or if NLP was applied to structured feedback or non–patient-generated content. Data were extracted and narratively synthesized regarding health care settings, NLP methods, and clinical applications.

**Results:**

From 4017 records, 52 studies met inclusion criteria. NLP was most commonly applied to UPF from secondary care settings (n=33) with fewer in primary (n=10) or community (n=5) care. Three NLP techniques were identified in the included studies: sentiment analysis (n=32), topic modeling (n=15), and text classification (n=7). Sentiment analysis was applied to explore associations between patient sentiment and health care provider characteristics, track emotional responses over time, and identify areas for improvement in health care delivery. Topic modeling, primarily using latent Dirichlet allocation algorithm, was used to uncover latent themes in patient feedback, compare patient experiences across different health care settings, and track changes in patient concerns over time. Text classification was used to categorize patient feedback into predefined topics. The association between NLP-derived insights and traditional health care quality metrics was limited, with few studies describing concrete clinical impacts resulting from their analyses.

**Conclusions:**

NLP has been applied to UPF across a number of contexts, primarily to identify features of health services or professionals that support good patient experience. The growth of research publications demonstrates an academic interest in these technologies, but there is little evidence these approaches are being used in clinical settings. Future research is required to assess how NLP may capture the nuance of health care interactions, align with existing quality metrics, and how it may be used to influence clinician behavior.

## Introduction

Patient experience is frequently used as an indicator of quality in health care systems [1]. Health care services that provide their patients with good experiences are more likely to retain patients; retention of patients both improves patient outcomes [2] and ensures the business viability of health services [3]. As such, health services make efforts to measure patient experience as part of quality assurance schemes.

To capture patient experiences, health care providers use various feedback mechanisms. Structured Patient Reported Experience Measures are commonly used to assess service quality [4] but are limited to the narrow concept that they intend to measure. In contrast, unstructured patient feedback (UPF) allows patients to describe their experiences of care without the restriction of preselected questions [5]. Health providers may use UPF in local quality improvement efforts, using suggestion boxes, testimonials, free-text feedback forms, and written patient complaints. By freely expressing their experiences, patients may identify issues that relate to high-quality and low-quality care that may not be measured in other ways [6,7].

While UPF provides rich insights, traditional methods of collecting, collating, and interpreting these data at large scales are time-consuming and resource-intensive [8]. This challenge has been amplified by the proliferation of online health care rating sites, which have become ubiquitous sources of patient experience and satisfaction data [9,10]. Unlike local feedback mechanisms, these platforms are not usually coordinated by the health service being reviewed, creating a vast, decentralized source of patient feedback. Studies associating patient experiences reported in web-based reviews and physician performance have demonstrated both poor [11,12] and good [13] correlation between patient experience and traditional performance metrics.

The proliferation of web-based reviews means that this is now a large data source that crosses a number of areas of health care [10,14]. The rate and scale at which new reviews are generated means that it would be infeasible for assessments of the state of different health to be made through conventional methodologies such as thematic analysis. As these data sources continue to expand, health care systems require more efficient approaches to extract meaningful insights from patient feedback.

Natural language processing (NLP) offers a solution by using machine learning and artificial intelligence algorithms to interpret text data [15] in a time-efficient and resource-efficient manner [16]. Beyond efficiency, NLP offers several advantages over conventional thematic analysis: it reduces human coding bias [17], enables reproducible analysis at scale [18], allows detection of subtle patterns that might not be apparent to human analysts [19], and permits longitudinal analysis of patient feedback over time to detect evolving trends [20]. NLP includes sentiment analysis, which assesses whether a text has an overall positive or negative sentiment to show people’s opinions, attitudes, and emotions [21], topic modeling, which determines the frequency and association of words within texts to develop topics of interest [22,23], and topic classification, where texts are classified to preselected topics.

Despite the growing application of NLP to patient feedback, there remains a significant gap in understanding how these methods are applied across different health settings. The extent to which NLP analysis of UPF is being used to drive quality improvement across different health care settings is currently unclear, limiting its potential impact on patient care. While NLP application to patient experience has been reported in contexts such as hospital care [24,25], general medical practice [26], and dentistry [27,28], the comprehensive landscape remains unmapped. A systematic review by Khanbhai et al [29] was performed in 2021 exploring the use of machine learning and NLP in patient experience feedback. The search conducted in this paper occurred in 2020, with 15 of 19 included papers identified in the years 2015‐2020. Preliminary literature searching has demonstrated a proliferation in studies exploring the use of NLP since the completion of this review, suggesting a more current and comprehensive assessment.

The aim of this scoping review is to investigate how NLP techniques are currently being used to interpret UPF across different health services. Objectives of this review were to identify the settings in which NLP has been used to interpret patient feedback, the purposes using NLP, and to understand whether the interpretation of patient feedback with NLP has been used to inform any changes in clinical practice or policy.

## Methods

### Eligibility Criteria

This scoping review follows the Joanna Briggs Institute methodology for scoping reviews [30]. As this review is looking at patient feedback provided across all types of health care, no specific population was defined. For inclusion, sources must have explored the use of NLP on UPF of a health service or provider. NLP was defined as computer-based algorithmic assessment of text, with or without training of probabilistic models. UPF was defined as any unstructured text or “free text,” written by a user of a health service to relate their outcomes or experience of that health service. The contexts explored in this review were health services, including, but not limited to, medicine, dentistry, physiotherapy, pharmacy, and ophthalmology. Papers that explored UPF that was attributable to a particular health service or provider were included. Primary sources of UPF in these papers may include in-clinic collection on comment cards, hospital websites online service rating platforms, or social media platforms.

Sources were excluded if coding and interpretation of UPF was performed by a human actor only. Studies using NLP on structured feedback, electronic health records, and chatbots were excluded because they represent fundamentally different data types and clinical applications. Structured feedback contains predefined response categories with limited expressive range, electronic health records contain clinician rather than patient language, and chatbot interactions represent dialogues rather than retrospective experiences.

Sources from the peer-reviewed and gray literature were considered. This included both experimental and quasi-experimental study designs, including randomized controlled trials, nonrandomized controlled trials, before and after studies, and interrupted time series studies. In addition, analytical observational studies including prospective and retrospective cohort studies, case-control studies, and analytical cross-sectional studies were considered for inclusion. Descriptive observational study designs, including case series, individual case reports, and descriptive cross-sectional studies, were considered for inclusion. In addition, systematic reviews that meet the inclusion criteria will also be considered, depending on the research question as a source of further papers for inclusion of further papers. Conference abstracts were also considered.

### Search Strategy

An initial limited search of MEDLINE and Embase was undertaken to identify papers. Keywords contained in the titles and abstracts of relevant papers, and the index terms used to describe the papers, were used to develop a full search strategy for MEDLINE, Embase, CINAHL, and Cochrane Database of Reviews (Multimedia Appendix 1). Reference lists of all included sources of evidence were screened for additional studies. Where studies were not available, authors were contacted directly to attempt to attain copies. To try and identify further peer-reviewed literature and gray literature, Google Scholar and Google were searched in advanced search function using keywords of “patients,” “patient feedback,” “health care,” “NLP,” “topic modeling,” “topic classification,” and “sentiment analysis.” Initial searches for gray literature identified limited information, primarily comprising opinion pieces on the promise of NLP rather than the impact of NLP on health services. In the interest of time and resources for the review, peer-reviewed sources were prioritized.

### Study or Source of Evidence Selection

All identified sources were collated in Endnote 20 (Clarivate Analytics) and duplicates were removed. Pilot screening was performed by 2 independent reviewers (AF and CL) on a sample of 20 papers to ensure consistent application of the inclusion and exclusion criteria. Following this, all titles and abstracts were screened by single authors using Rayyan [31]. Full texts of shortlisted papers were assessed in detail against the inclusion and exclusion criteria by 2 independent reviewers (AF and CL). Disagreements were resolved through regular meetings, with a third reviewer (MB) acting as a tiebreaker. Reasons for exclusion of sources of evidence at full text that did not meet the inclusion criteria were recorded and are reported in the PRISMA (Preferred Reporting Items for Systematic reviews and Meta-Analyses) flow diagram. While formal critical appraisal was not conducted as per scoping review methodology, we assessed the reliability of included studies through research team discussions during data extraction meetings to identify methodological concerns. We considered the peer-review process of the journals in which studies were published as a quality filter. Additionally, we considered journal accessibility to ensure that our review captured studies that would be readily available to health care practitioners and researchers interested in implementing NLP approaches.

### Data Extraction

Data were independently extracted by 3 reviewers (AF, CL, and MB) using a data extraction spreadsheet developed by the reviewers (Multimedia Appendix 2). Heterogeneity of methods, contexts, and reporting meant that quantitative comparison was not possible. Qualitative assessment and narrative summaries of reviews were prepared in the extraction tool. The research team conducted an internal review of the extraction process through regular meetings to ensure consistency. Following initial extraction, further elements to the form were added to record any performance metrics associated with the NLP methods. As a scoping review, critical appraisal of individual pieces of evidence was not carried out.

## Results

### Search Results

Five databases were searched in February 2024, yielding 4017 records: MEDLINE (n=675), Embase (n=2286), CINAHL (n=1024), Cochrane Database of Reviews (n=3), and Google Scholar (n=29). After removing duplicates, 3433 were screened by 2 independent reviewers (AF and CL), resulting in 3052 exclusions. An additional 283 records were marked as “maybe,” and 64 records had conflicts that required discussion. Following discussions with a third reviewer (MB), 92 records were included while 3341 were excluded.

**Figure 1.**
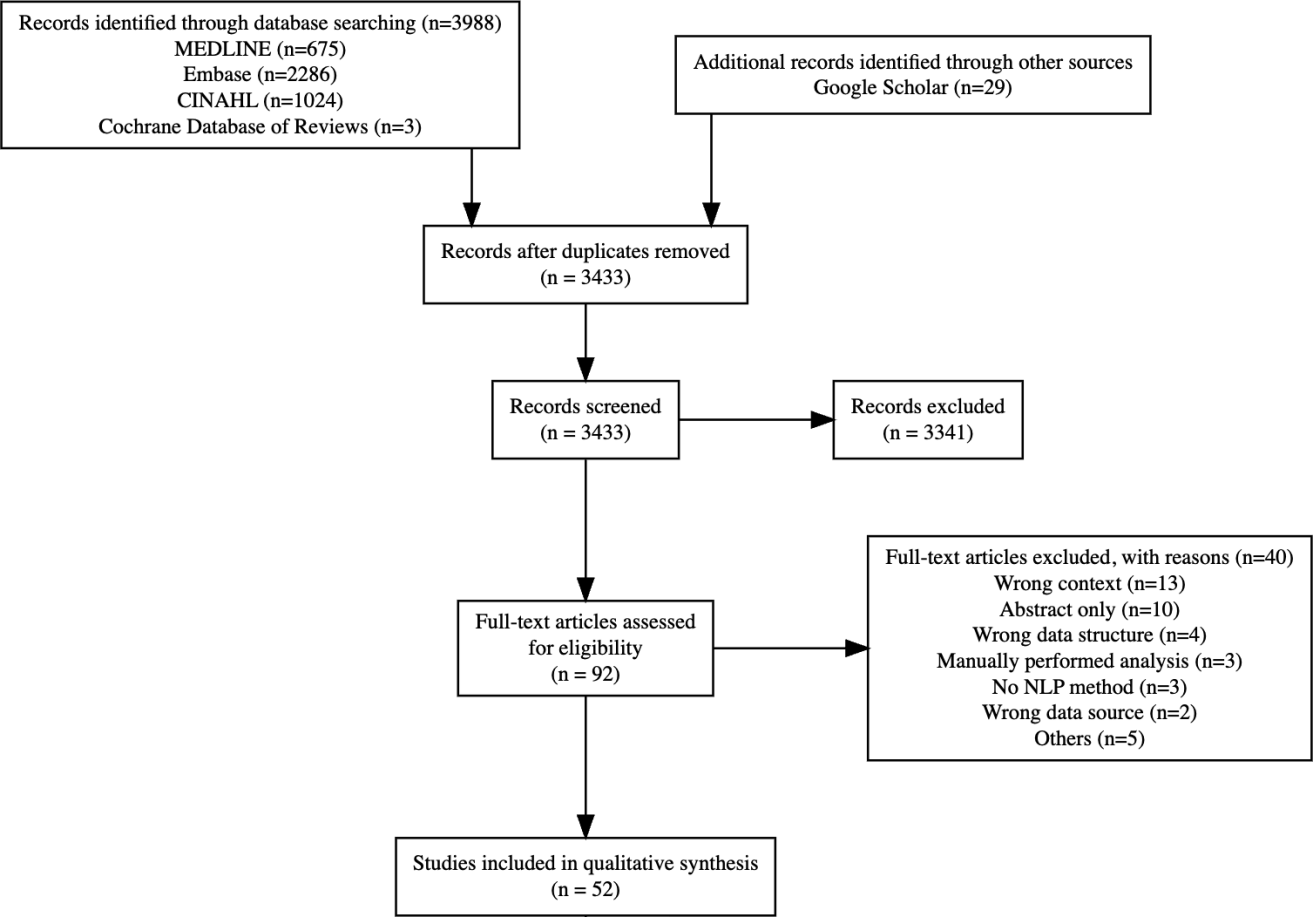
PRISMA (Preferred Reporting Items for Systematic reviews and Meta-Analyses) flow diagram. NLP: natural language processing.

Full-text screening of these 92 records resulted in the inclusion of 52 studies and the exclusion of 40 studies based on the predefined inclusion and exclusion criteria (Figure 1). The summary of the included studies is reported in Multimedia Appendix 3 [7,16,21,27,32-70].

### Health Care Settings in Which NLP Has Been Used to Assess UPF

Seven research papers were associated with health services in English-speaking nations (United States=3, United Kingdom=4). Ten studies were carried out in non–English-speaking nations (Iran=3, India=2, China=2, South Korea=1, Spain=1, and Netherlands=1). Three broad categories of NLP were used: sentiment analysis, topic modeling, or text classification. The distribution of reviewed papers based on their health care setting (primary, secondary, or community care) and the specific NLP method used are shown in Table 1. Note that the total count in the table is greater than the number of reviewed papers, as some papers use more than 1 method in their analysis.

**Table 1.** Distribution of natural language processing review papers by health care setting and natural language processing method (note: some studies used multiple methods).

	Sentiment analysis, n	Topic modeling, n	Text classification, n
Primary care	7	1	2
Secondary care	21	9	3
Community	2	3	0
Unspecified	2	2	2
Total	32	15	7

### Applications of NLP

#### Sentiment Analysis

Sentiment analysis was used in 32 of the 52 studies considered. VADER [71], a “stock” open-source sentiment analysis tool, was used in 11 studies [21,32-41]. Basic machine learning algorithms were used in 8 of the reviewed papers for sentiment analysis, including support vector machine learning [42-46], Naïve Bayes [44-47], and Decision Tree [45,48]. Advanced neural networks were used in 3 of the reviewed papers and included the Keras library [16], recurrent neural networks [49], and convolutional neural networks [50]. Third-party paid sentiment analysis services used included Crystalfeel [51,52], Baidu [53], Tencent [54], IBM Watson [55], and the Press Ganey Associates’ NLP tools for surveys and feedback forms [56].

A number of studies used sentiment analysis to explore the associations between patient sentiment and demographic factors of clinicians, such as their age and their geographical location. Studies examining web-based reviews of otolaryngologists [32], spine surgeons [35], neurosurgeons [38], urologists [40], and psychiatrists [39] found that younger practitioners received higher sentiment scores. Location was shown to be a significant factor in sentiment scores of neurosurgeons [38], psychiatrists [39], and otolaryngologists [32]. Several studies in this review demonstrate a strong correlation between sentiment scores derived from patient reviews and the star ratings given to health care providers [16,43,50,55].

Word frequency analysis was often used alongside sentiment to characterize patient sentiments in their interactions with health care. By analyzing the language used in web-based reviews and comments, researchers can uncover common themes, concerns, and areas for improvement in health care delivery [32]. For example, Park et al [39] found that, for psychiatrists, positive reviews mentioned “time” and “caring,” whereas negative reviews mentioned “medication.” Pain management was identified as a significant driver of patient satisfaction across various surgical specialties, including hand surgery [33], scoliosis surgery [34], and spine surgery [35]. Gour and Kumari [57] used fuzzy sentiment analysis and word frequency analysis to identify trust and fear as components of positive and negative reviews. Nawab et al [16] combined sentiment analysis with word frequency to associate hospital room conditions and discharge processes as markers of quality.

Sentiment analysis was also used to track patients’ emotional responses to health care services over time. Shah et al [52] used aspect-based sentiment analysis to track changes in patient sentiment and topics of concern expressed in web-based reviews over the course of the COVID-19 pandemic, showing that fear, anger, and sadness in the early stages of the pandemic gradually shifted toward more positive sentiments. Similarly, Li et al [53] used sentiment and word frequency analysis to investigate changes in doctor-patient relationships during COVID-19, noting a shift in the focus of negative comments from personal attitudes to administrative issues. Further applications of sentiment analysis include assessment of transitions of care and continuity [58]. Hu et al [54] monitored public perceptions on health care services using social media data in China. The findings indicated that the doctor-patient relationship category had the highest proportion of negative contents, followed by service efficiency and nursing service.

#### Topic Modeling

Topic modeling methods were applied in 15 of the 52 studies. Topic modeling is an unsupervised machine learning technique that can scan large volumes of text to identify latent topics based on word co-occurrence [72]. Yazdani et al [59] used topic modeling on feedback of hospitalized patients with cancer, identifying dissatisfaction with appointment booking services and positive experiences with staff and chemotherapy. Stokes et al [60] analyzed narrative themes in web-based reviews of mental health facilities, identifying that caring staff and nonpharmacologic treatment modalities were positively correlated with high ratings, while issues such as safety and abuse and poor communication were linked to negative reviews. Agarwal et al [58] compared web-based ratings of patient experiences between emergency departments and urgent care centers, finding comfort, professionalism, and staff interactions being key themes in 5-star reviews for both types of facilities.

Lin et al [27] applied topic modeling to web-based reviews of dental care, finding that higher ratings were associated with female dentists, dentists at a younger age, and those whose patients experienced a short wait time. They also identified several topics that corresponded to Consumer Assessment of Healthcare Providers and Systems (CAHPS) measures [73], including discomfort (eg, painful or painless root canal or deep cleaning) and ethics (eg, high-pressure sales and unnecessary dental work) [27].

Latent Dirichlet allocation (LDA) is a type of topic modeling that assumes that each document is a mixture of a small number of topics and that each word’s presence is attributable to one of the document’s topics [72]. Ranard et al [7] used LDA to identify topics in Yelp reviews of hospitals, demonstrating that reviews covered more topics than the Hospital Consumer Assessment of Healthcare Providers and Systems (HCAHPS) survey [74], including the cost of hospital visits, insurance, billing, and the quality of nursing and staff. LDA analysis of pharmacy Yelp reviews by Lester and Chui [61] revealed 4 key topics: prescription wait times, staff helpfulness, store environment, and medication filling issues. Pearson correlations showed that wait times and filling issues negatively correlated with ratings, while staff helpfulness and store environment showed positive correlations [61].

Extensions and modifications to LDA were used in a number of papers: Shah et al [51] developed Topic Coherence-Based LDA to improve the interpretability of topics by measuring the semantic similarity between high-scoring words in a topic. Topic Coherence-Based LDA was used to identify emerging and fading topics in patient web-based reviews during the early wave of the COVID-19 pandemic showing an increased focus on treatment experiences, policy implementation, and mental health developed over time [51]. In a further study, Shah et al [52] used dynamic topic modeling to investigate the dynamics of public concerns and sentiments expressed in 2018, 2019, and 2020. This showed that topics shifted from general health care issues to pandemic-specific concerns such as virus transmission, travel restrictions, and government countermeasures. Sentiments initially became more negative, with anger as the dominant emotion [52].

Gibbs Sampling Dirichlet Mixture Model, a modified version of LDA that assumes that each document has only 1 topic, making it more efficient for dealing with short texts [75]. Serrano-Guerrero et al [21] used Gibbs Sampling Dirichlet Mixture Model to group sentences from patient opinions and identify the most frequent topics such as coordination, scheduling appointments, order queue, community support, and so on, related to nurses and doctors in different health care categories (high-risk disease, low-risk disease, and infectious disease).

Nonnegative matrix factorization factorizes a matrix into 2 nonnegative matrices [76] for dimensionality reduction and feature extraction and has been reported to provide more interpretable topics than LDA [77]. Langerhuizen et al [55] used nonnegative matrix factorization to identify the 50 most frequently occurring 3-word combinations (eg, poor bedside manner, office staff rude, waited lobby hour) in web-based reviews of orthopedic surgeons and office, identifying themes such as logistics, care and compassion, trust, recommendation, and customer service as important elements of quality. Tones of joy and confidence were associated with higher ratings. Sadness and tentative tones were associated with lower ratings.

#### Text Classification

Seven studies conducted text classification analysis. Text classification uses machine learning to assign predefined categories or labels to textual data. This process involves training a model on a labeled dataset of texts that provides the “ground truth” from which the model can learn to predict the labels of new, unseen text [78]. This review identified 2 instances where UPF was used to train NLP text classifiers.

Khanbhai et al [42] applied supervised learning algorithms to categorize patient feedback. This involves training models on labeled datasets to classify new, unlabeled data accurately. The study also uses topic classification tools such as the KoNstanz Information MinEr platform for qualitative content analysis [79]. This approach helps in systematically categorizing patient concerns into distinct topics, providing a structured overview of patient feedback.

Similarly, He et al [62] integrate both supervised and unsupervised machine learning approaches. Initially, a set of reviews is manually coded to identify major themes, a process known as qualitative coding [62]. These coded data then serve as the training set for supervised machine learning algorithms, enabling the classifiers to generalize the identified themes across the entire dataset. Moreover, the study uses unsupervised learning techniques, such as word clustering using the k-means algorithm, to identify fine-grained aspects of patient concerns.

### Changes to Clinical Practices Reported

While almost all of the studies examined stated the potential of NLP for UPF to inform clinical practices and changes in behaviors, few concrete changes to clinical practice were reported. Parikh et al [63] describe how NLP is used to analyze patient feedback from magnetic resonance imaging scans to identify potential care issues, allowing appropriate clinical teams to be notified and intervene before problems escalate. Nawab et al [16] demonstrated how NLP of patient experience comments allowed for quick identification of negative markers of patient experience within a hospital setting, identifying climate control and temperature of waiting rooms as a factor that could be easily modified. Menendez et al [56] used NLP of negative reviews to identify the main sources of complaints in an orthopedic hospital, showing that improvements in the quality of patient rooms would likely yield the greatest improvement in patient experience. Khanbhai et al [42] describe how the use of NLP of free-text comment of friend and family test results in 4 hospital trusts in the United Kingdom can be used to facilitate rapid action on feedback; while no specific examples of actions implemented were given, the benefit of shifting resource from manual analysis of reviews to implementation of quality improvement actions was highlighted. A similar benefit of time saving of human evaluators of patient feedback was also reported by Khaleghparast et al [48].

Cammel et al [64] used sentiment analysis, topic modeling, and prioritization factorization to develop top 5 rankings for areas needing improvement and ongoing monitoring within different hospital environments, translating their findings into actionable priorities for hospital improvement initiatives.

Alemi and Jasper [80] used NLP alongside traditional summarization of text to allow managers of departments to assess the quality of care across domains related to the CAHPs survey. This was intended to allow for targeted improving of quality improvement activities in different clinical contexts within a hospital.

## Discussion

### Principal Findings

While NLP has been routinely used in business and the service industry to analyze customer reviews for service improvement [37,65], health care has been slower to adopt these technologies. This review demonstrates a growing application of NLP to patient feedback in health care but a significant gap in knowledge of how best to translate these findings into actions that will improve a patient experience and outcomes. Our scoping review significantly updates and builds on the work of Khanbhai et al [29], whose 2021 systematic review analyzed 19 studies on NLP and machine learning applications for patient experience feedback published until December 2019. Although the review by Khanbhai et al offered valuable insights into this emerging field, our review reveals rapid growth, increasing the number of studies from 19 to 52 within a short period and reflecting heightened academic interest in NLP for patient feedback. Despite this progress, our review confirms that a research-to-practice gap remains, underscoring the need for future work to focus on practical implementation rather than solely on technical feasibility.

Sentiment analysis emerged as the most commonly used form of NLP for UPF in this review. When used alongside word frequency analysis or topic modeling, sentiment analysis may indicate reasons for satisfaction or dissatisfaction with care. The technology has demonstrated practical value in several contexts, including identifying environmental factors affecting patient experience [16], or areas of patient dissatisfaction to inform potential changes to practice [16]. These factors may not have been picked up in traditional measures of patient experience or health care quality that are limited by the content of preconceived questions of researchers or clinicians. However, this review demonstrates a proliferation of studies using simple sentiment analysis tools such as VADER to relate patient satisfaction to demographic factors of clinicians such as their age or gender. Such simplistic analyses do not offer much benefit in terms of assessing the quality of clinicians’ decision-making or clinical skill; greater interrogation of underlying factors is indicated. Overreliance on simple tools for sentiment analysis may not capture the complexity of health care experiences. Many interactions with health services are likely to cause a degree of physical or mental discomfort, even if overall outcomes and experiences are good, leading patients to express mixed emotions about their care. More sophisticated approaches using advanced neural networks show promise in capturing these nuances, as demonstrated by Gui and He [50], whose convolutional neural networks outperformed traditional methods in analyzing sentiment subtleties in health care reviews. Nawab et al [16] used neural network approaches effectively to identify specific aspects of patient experience. Recent developments in transformer-based models such as BERT [81] and large language models [82] offer potential improvements through their ability to better understand health care–specific terminology and context, although their application to patient feedback analysis remains limited in the current literature. Future developments for sentiment analysis should focus on health care–specific models that account for the unique context of medical experiences, integration with existing quality improvement frameworks, and validation studies comparing automated sentiment analysis against traditional patient experience measures.

Topic modeling emerged as a practical tool for health care administrators to efficiently process large volumes of patient feedback, serving primarily administrative and public relations purposes rather than driving clinical improvements. Studies demonstrate its use in rapidly identifying operational issues such as appointment-booking problems in cancer care [59], service quality in mental health facilities [60], and comparing emergency and urgent care services [58]. LDA can reveal insights and latent themes in UPF that would not be identified in traditional surveys, such as billing concerns and wait times [7,61]. There is limited evidence of these insights translating into concrete clinical or service changes. This suggests a gap between the potential of topic modeling for health care quality improvement and its current practical applications in health care settings. If topic modeling were to be used more widely for quality improvement purposes, the limitations of this as a technology and the decisions made by researchers in developing models must be appropriately recorded and reported. As topical modeling is an unsupervised machine learning technique, it requires a process of trial and error to yield meaningful results. Researchers must make subjective judgments about the number of topics to extract and interpret the resulting topic clusters, which introduces a potential for bias. The manual labeling of topics identified through LDA, as demonstrated in the studies by Lin et al [27] and Stokes et al [60], inevitably involves some degree of subjective decision-making. Transparent reporting of the philosophical positions of the researchers labeling and interpreting the topics through reflexivity statements, reinterpretation of data by different research groups, or the involvement of patient and practitioner stakeholder groups in the development of models may help improve the validity of these techniques in health care research.

When considering the effectiveness of different NLP approaches for analyzing UPF, it is important to note that performance varies significantly based on multiple factors including data characteristics (volume, length of comments, and language complexity), health care context, preprocessing techniques used, and the specific objectives of the analysis. Methods that demonstrate high performance in one setting may not necessarily translate to others with the same effectiveness. Rather than identifying a single ‘best’ approach, health care organizations should consider their specific requirements, available resources, and the nature of their patient feedback data when selecting appropriate NLP methods. This context-dependent performance highlights the need for careful method selection and evaluation when implementing NLP solutions for patient feedback analysis.

A significant issue is the limited association between NLP-derived insights and traditional concepts of health care quality. Many established quality metrics in health care are based on clinical outcomes, process measures, and standardized patient surveys such as HCAHPS and CAHPS. Several studies aimed to demonstrate the validity of their models in assessing quality and performance of clinicians by demonstrating correlations between sentiment scores of unstructured and conventional star ratings used to rate an experience as a whole [16,43,50,55]; the relationship to broader health care quality metrics remains underexplored. UPF analyzed by NLP techniques is a rich source of inpatient perspectives and captures themes that extend beyond traditional survey measures. For example, the analysis of hospital Yelp reviews by Ranard et al [7] revealed that both topics contained within the HCAHPS and those not, including cost of hospital visits, insurance, and billing issues, could be identified through NLP of UPF. Similarly, the analysis of dental care reviews by Lin et al [27] identified additional quality indicators such as discomfort during procedures and ethical concerns about unnecessary treatments. This broader scope of patient feedback, while valuable, may not easily align with traditional metrics. This misalignment between NLP-derived insights and established quality frameworks can lead to skepticism among health care professionals and policy makers about the validity and use of these approaches, despite their potential to capture important aspects of patient experience that traditional metrics might miss.

The scarcity of papers describing tangible clinical outcomes resulting from NLP analyses suggests that academic rather than translational impact has been the driving force behind research so far. This gap between academic research and clinical practice raises questions about the practical use of these approaches in improving health care delivery. The research community should now look toward the implementation of science methods to assess how best to use NLP to enact real-world change. In order for feedback interventions to be effective and affect changes in clinical behaviors, they must be targeted, relevant, and deemed as trustworthy and valid by clinicians [83]. Greater understanding of the perceived validity of insights developed from NLP must be gained prior to its widespread adoption. Study designs for NLP research should demonstrate clear pathways to clinical application and include measures of clinical impact. Further collaboration between NLP researchers and health care providers should be encouraged to ensure that research questions address real-world clinical needs. Where clinical impacts are described, it is important to consider whether these occur at the policy maker level, institutional level, or individual clinician level.

### Limitations

This scoping review has several limitations that should be considered when interpreting its findings. First, the review was limited to English language publications, potentially excluding relevant studies published in other languages and introducing a language bias. The rapid evolution of NLP technologies means that some of the most recent advancements may not be fully represented in the published literature included in this review. The heterogeneity of NLP techniques and health care settings made it challenging to draw direct comparisons between studies or to conduct a quantitative meta-analysis. Additionally, the review did not assess the quality of individual studies, which is typical for scoping reviews, but may limit the ability to evaluate the robustness of the reported findings. Finally, the focus on published literature may have introduced publication bias, potentially overlooking unpublished work or ongoing projects in the field of NLP application in health care.

### Conclusions

While NLP techniques offer promising avenues for enhancing patient-centered care and quality improvement in health care, significant work remains to translate these technological advancements into meaningful clinical outcomes. Despite limitations, this review provides a comprehensive overview of the current state of NLP applications in analyzing UPF across various health care settings. While NLP techniques demonstrated potential in analyzing large volumes of patient feedback efficiently, there was limited evidence of these insights translating into tangible clinical impacts or quality improvement initiatives. To realize the potential for NLP of UPF, future research must bridge the gap between academic interest and clinical impact. This calls for closer collaboration between NLP researchers and health care providers, study designs that demonstrate clear pathways to clinical application, and more effective methods for disseminating insights to health care professionals.

## Supplementary material

10.2196/72853 Multimedia Appendix 1 Search strategy.

10.2196/72853 Multimedia Appendix 2 Data extraction tool.

10.2196/72853 Multimedia Appendix 3 Summary tables.

10.2196/72853 Checklist 1 PRISMA-ScR (Preferred Reporting Items for Systematic Reviews and Meta-Analyses extension for Scoping Reviews) checklist.

## Abbreviations

CAHPS: Consumer Assessment of Healthcare Providers and Systems
HCAHPS: Hospital Consumer Assessment of Healthcare Providers and Systems
LDA: latent Dirichlet allocation
NLP: natural language processing
PRISMA: Preferred Reporting Items for Systematic Reviews and Meta-Analyses
PRISMA-ScR: Preferred Reporting Items for Systematic Reviews and Meta-Analyses extension for Scoping Reviews
UPF: unstructured patient feedback

## References

[1] Burt J, Campbell J, Abel G (2017). Improving patient experience in primary care: a multimethod programme of research on the measurement and improvement of patient experience. Programme Grants Appl Res.

[2] Fenton JJ, Jerant AF, Bertakis KD, Franks P (2012). The cost of satisfaction: a national study of patient satisfaction, health care utilization, expenditures, and mortality. Arch Intern Med.

[3] Kessler DP, Mylod D (2011). Does patient satisfaction affect patient loyalty?. Int J Health Care Qual Assur.

[4] Gleeson H, Calderon A, Swami V, Deighton J, Wolpert M, Edbrooke-Childs J (2016). Systematic review of approaches to using patient experience data for quality improvement in healthcare settings. BMJ Open.

[5] Lu Z, Sim JA, Wang JX (2021). Natural language processing and machine learning methods to characterize unstructured patient-reported outcomes: validation study. J Med Internet Res.

[6] Greaves F, Ramirez-Cano D, Millett C, Darzi A, Donaldson L (2013). Harnessing the cloud of patient experience: using social media to detect poor quality healthcare. BMJ Qual Saf.

[7] Ranard BL, Werner RM, Antanavicius T (2016). Yelp reviews of hospital care can supplement and inform traditional surveys of the patient experience of care. Health Aff (Millwood).

[8] Wagland R, Recio-Saucedo A, Simon M (2016). Development and testing of a text-mining approach to analyse patients’ comments on their experiences of colorectal cancer care. BMJ Qual Saf.

[9] Reimann S, Strech D (2010). The representation of patient experience and satisfaction in physician rating sites. A criteria-based analysis of English- and German-language sites. BMC Health Serv Res.

[10] Boylan AM, Williams V, Powell J (2020). Online patient feedback: a scoping review and stakeholder consultation to guide health policy. J Health Serv Res Policy.

[11] Daskivich TJ, Houman J, Fuller G, Black JT, Kim HL, Spiegel B (2018). Online physician ratings fail to predict actual performance on measures of quality, value, and peer review. J Am Med Inform Assoc.

[12] Chen J, Presson A, Zhang C, Ray D, Finlayson S, Glasgow R (2018). Online physician review websites poorly correlate to a validated metric of patient satisfaction. J Surg Res.

[13] Griffiths A, Leaver MP (2018). Wisdom of patients: predicting the quality of care using aggregated patient feedback. BMJ Qual Saf.

[14] Trehan SK, Daluiski A (2016). Online patient ratings: why they matter and what they mean. J Hand Surg Am.

[15] Liddy ED (2001). Encyclopedia of Library and Information Science, 2nd ed.

[16] Nawab K, Ramsey G, Schreiber R (2020). Natural language processing to extract meaningful information from patient experience feedback. Appl Clin Inform.

[17] Guda N (2023). Analyzing the extent to which gender bias exists in news articles using natural language processing techniques. J Stud Res.

[18] Belz A (2022). A metrological perspective on reproducibility in NLP*. Comput Linguist Assoc Comput Linguist.

[19] Alexander G, Bahja M, Butt GF (2022). Automating large-scale health care service feedback analysis: sentiment analysis and topic modeling study. JMIR Med Inform.

[20] Farrell MJ, Brierley L, Willoughby A, Yates A, Mideo N (2022). Past and future uses of text mining in ecology and evolution. Proc Biol Sci.

[21] Serrano-Guerrero J, Bani-Doumi M, Chiclana F, Romero FP, Olivas JA (2024). How satisfied are patients with nursing care and why? A comprehensive study based on social media and opinion mining. Inform Health Soc Care.

[22] Scharkow M (2013). Thematic content analysis using supervised machine learning: an empirical evaluation using German online news. Qual Quant.

[23] Canini K, Shi L, Griffiths T Online inference of topics with Latent Dirichlet Allocation.

[24] van Buchem MM, Neve OM, Kant IMJ, Steyerberg EW, Boosman H, Hensen EF (2022). Analyzing patient experiences using natural language processing: development and validation of the Artificial Intelligence Patient Reported Experience Measure (AI-PREM). BMC Med Inform Decis Mak.

[25] A. Rahim AI, Ibrahim MI, Musa KI, Chua SL (2021). Facebook reviews as a supplemental tool for hospital patient satisfaction and its relationship with hospital accreditation in Malaysia. Int J Environ Res Public Health.

[26] Gray BM, Vandergrift JL, Gao GG, McCullough JS, Lipner RS (2015). Website ratings of physicians and their quality of care. JAMA Intern Med.

[27] Lin Y, Hong YA, Henson BS (2020). Assessing patient experience and healthcare quality of dental care using patient online reviews in the United States: mixed methods study. J Med Internet Res.

[28] Ponathil A, Khasawneh A, Byrne K, Chalil Madathil K (2021). Factors affecting the choice of a dental care provider by older adults based on online consumer reviews. IISE Trans Healthc Syst Eng.

[29] Khanbhai M, Anyadi P, Symons J, Flott K, Darzi A, Mayer E (2021). Applying natural language processing and machine learning techniques to patient experience feedback: a systematic review. BMJ Health Care Inform.

[30] Peters MDJ, Godfrey C, McInerney P, Munn Z, Tricco AC, Khalil H, Aromataris E, Munn Z (2020). JBI Manual for Evidence Synthesis.

[31] Intelligent systematic review. Rayyan.

[32] Vasan V, Cheng CP, Lerner DK, Vujovic D, van Gerwen M, Iloreta AM (2023). A natural language processing approach to uncover patterns among online ratings of otolaryngologists. J Laryngol Otol.

[33] Tang JE, Arvind V, White CA (2023). Using sentiment analysis to understand what patients are saying about hand surgeons online. Hand (N Y).

[34] Tang JE, Arvind V, White CA, Dominy C, Kim JS, Cho SK (2022). What are patients saying about you online? A sentiment analysis of online written reviews on Scoliosis Research Society surgeons. Spine Deform.

[35] Tang JE, Arvind V, Dominy C, White CA, Cho SK, Kim JS (2023). How are patients reviewing spine surgeons online? A sentiment analysis of physician review website written comments. Global Spine J.

[36] Tang J, Arvind V, White CA, Dominy C, Cho S, Kim JS (2023). How are patients describing you online? A natural language processing driven sentiment analysis of online reviews on CSRS surgeons. Clin Spine Surg.

[37] Sewalk KC, Tuli G, Hswen Y, Brownstein JS, Hawkins JB (2018). Using Twitter to examine web-based patient experience sentiments in the United States: longitudinal study. J Med Internet Res.

[38] Quinones A, Tang J, Vasan V, Li T, Li A, Durbin J (2022). Trends in online patient perspectives of neurosurgeons: a sentiment analysis. J Neurosurg.

[39] Park SH, Cheng CP, Buehler NJ, Sanford T, Torrey W (2023). A sentiment analysis on online psychiatrist reviews to identify clinical attributes of psychiatrists that shape the therapeutic alliance. Front Psychiatry.

[40] Levy M, Tang JE, Chin CP (2023). Sentiment analysis of online written reviews for a national urology cohort illustrates important factors for patient satisfaction. Journal of Urology.

[41] Pandey AR, Seify M, Okonta U, Hosseinian-Far A (2023). Advanced sentiment analysis for managing and improving patient experience: application for general practitioner (GP) classification in Northamptonshire. Int J Environ Res Public Health.

[42] Khanbhai M, Warren L, Symons J (2022). Using natural language processing to understand, facilitate and maintain continuity in patient experience across transitions of care. Int J Med Inform.

[43] Jiménez-Zafra SM, Martín-Valdivia MT, Molina-González MD, Ureña-López LA (2019). How do we talk about doctors and drugs? Sentiment analysis in forums expressing opinions for medical domain. Artif Intell Med.

[44] Huppertz JW, Otto P (2018). Predicting HCAHPS scores from hospitals’ social media pages: a sentiment analysis. Health Care Manage Rev.

[45] Alemi F, Torii M, Clementz L, Aron DC (2012). Feasibility of real-time satisfaction surveys through automated analysis of patients’ unstructured comments and sentiments. Qual Manag Health Care.

[46] Agrawal S, Jain SK, Sharma S, Khatri A (2022). COVID-19 public opinion: a Twitter healthcare data processing using machine learning methodologies. Int J Environ Res Public Health.

[47] Hawkins JB, Brownstein JS, Tuli G (2016). Measuring patient-perceived quality of care in US hospitals using Twitter. BMJ Qual Saf.

[48] Khaleghparast S, Maleki M, Hajianfar G (2023). Development of a patients’ satisfaction analysis system using machine learning and lexicon-based methods. BMC Health Serv Res.

[49] Kao M, Leong M, Prasad R (2015). (244) Stanford Patient Experience Questionnaire (SPEQ): machine-mediated classification of patient experience feedback using natural language processing. J Pain.

[50] Gui L, He Y (2021). Understanding patient reviews with minimum supervision. Artif Intell Med.

[51] Shah AM, Yan X, Qayyum A, Naqvi RA, Shah SJ (2021). Mining topic and sentiment dynamics in physician rating websites during the early wave of the COVID-19 pandemic: machine learning approach. Int J Med Inform.

[52] Shah AM, Naqvi RA, Jeong OR (2021). Detecting topic and sentiment trends in physician rating websites: analysis of online reviews using 3-wave datasets. Int J Environ Res Public Health.

[53] Li J, Pang PCI, Xiao Y, Wong D (2022). Changes in doctor-patient relationships in China during COVID-19: a text mining analysis. Int J Environ Res Public Health.

[54] Hu G, Han X, Zhou H, Liu Y (2019). Public perception on healthcare services: evidence from social media platforms in China. Int J Environ Res Public Health.

[55] Langerhuizen DWG, Brown LE, Doornberg JN, Ring D, Kerkhoffs G, Janssen SJ (2021). Analysis of online reviews of orthopaedic surgeons and orthopaedic practices using natural language processing. J Am Acad Orthop Surg.

[56] Menendez ME, Shaker J, Lawler SM, Ring D, Jawa A (2019). Negative patient-experience comments after total shoulder arthroplasty. J Bone Joint Surg Am.

[57] Gour A, Kumari S (2021). A 360-degree view of a hospital by analysing patient’s online reviews using fuzzy sentiment analysis. J Health Manag.

[58] Agarwal AK, Mahoney K, Lanza AL (2019). Online ratings of the patient experience: emergency departments versus urgent care centers. Ann Emerg Med.

[59] Yazdani A, Shamloo M, Khaki M, Nahvijou A (2023). Use of sentiment analysis for capturing hospitalized cancer patients’ experience from free-text comments in the Persian language. BMC Med Inform Decis Mak.

[60] Stokes DC, Kishton R, McCalpin HJ (2021). Online reviews of mental health treatment facilities: narrative themes associated with positive and negative ratings. Psychiatr Serv.

[61] Lester C, Chui M (2017). Evaluating the patient experience at community pharmacies using yelp reviews. J Am Pharm Assoc.

[62] He L, He C, Wang Y, Hu Z, Zheng K, Chen Y (2020). What do patients care about? Mining fine-grained patient concerns from online physician reviews through computer-assisted multi-level qualitative analysis. AMIA Annu Symp Proc.

[63] Parikh P, Klanderman M, Teck A (2024). Effects of Patient Demographics and Examination Factors on Patient Experience in Outpatient MRI Appointments. J Am Coll Radiol.

[64] Cammel SA, De Vos MS, van Soest D (2020). How to automatically turn patient experience free-text responses into actionable insights: a natural language programming (NLP) approach. BMC Med Inform Decis Mak.

[65] Jung Y, Hur C, Jung D, Kim M (2015). Identifying key hospital service quality factors in online health communities. J Med Internet Res.

[66] Almorox EG, Stokes J, Morciano M (2022). Has COVID-19 changed carer’s views of health and care integration in care homes? A sentiment difference-in-difference analysis of on-line service reviews. Health Policy.

[67] Graves RL, Goldshear J, Perrone J (2018). Patient narratives in Yelp reviews offer insight into opioid experiences and the challenges of pain management. Pain Manag.

[68] Chekijian S, Li H, Fodeh S (2021). Emergency care and the patient experience: using sentiment analysis and topic modeling to understand the impact of the COVID-19 pandemic. Health Technol (Berl).

[69] Chan E, Korotkaya Y, Osadchiy V, Sridhar A (2022). Patient experiences at California crisis pregnancy centers: a mixed-methods analysis of online crowd-sourced reviews, 2010-2019. South Med J.

[70] Rajagopalan D, Thomas J, Ring D, Fatehi A (2022). Quantitative patient-reported experience measures derived from natural language processing have a normal distribution and no ceiling effect. Qual Manag Health Care.

[71] Hutto C, Gilbert E VADER: a parsimonious rule-based model for sentiment analysis of social media text.

[72] Blei DM, Ng AY, Jordan MI (2003). Latent Dirichlet allocation. J Mach Learn Res.

[73] (2024). CAHPS measures of patient experience. Agency for Healthcare Research and Quality.

[74] HCAHPS: patients’ perspectives of care survey. Centers for Medicare & Medicaid Services.

[75] Yin J, Wang J A dirichlet multinomial mixture model-based approach for short text clustering.

[76] Lee DD, Seung HS (1999). Learning the parts of objects by non-negative matrix factorization. Nature New Biol.

[77] Chuang J, Manning CD, Heer J Termite: visualization techniques for assessing textual topic models.

[78] Li Q, Peng H, Li J (2022). A Survey on Text Classification: From Traditional to Deep Learning. ACM Trans Intell Syst Technol.

[79] KNIME analytics platform. KNIME.

[80] Alemi F, Jasper H (2014). An alternative to satisfaction surveys: let the patients talk. Qual Manag Health Care.

[81] Koroteev MV (2021). BERT: a review of applications in natural language processing and understanding. arXiv.

[82] Zhao WX, Zhou K, Li J, Tang T, Wang X, Hou Y (2023). A survey of large language models. arXiv.

[83] Brown B, Gude WT, Blakeman T (2019). Clinical Performance Feedback Intervention Theory (CP-FIT): a new theory for designing, implementing, and evaluating feedback in health care based on a systematic review and meta-synthesis of qualitative research. Implement Sci.

