# Supplementary material for: The Use of Natural Language Processing to Interpret Unstructured Patient Feedback on Health Services: Scoping Review
Source: J Med Internet Res. 2025 Aug 14;27:e72853. doi: 10.2196/72853 (PMC12352804; doi:10.2196/72853)
Supplement: Multimedia Appendix 1 [file jmir-v27-e72853-s001.docx]

**Multimedia Appendix 1**: Search strategy

Full search strategy for the databases (MEDLINE, EMBASE, CINHAL, and Cochrane Database of Reviews). The search is built on the following concepts:

Public - lines 1 to 5

Feedback - lines 6 to 16

Healthcare setting – lines 17 to 22

NLP – line 23

1. Patients.mp. or exp Patients/

2. exp Consumer Behavior/

3. service adj3 user.mp.

4. client

5. public.mp.

6. exp Public Opinion/

7. exp Patient Satisfaction/

8. exp Patient-Centered Care/

9. exp Physician-Patient Relations/

10. exp Quality Assurance, Health Care/

11. exp Outcome Assessment, Health Care/

12. exp "Outcome and Process Assessment, Health Care"/ or exp Patient Outcome Assessment/

13. exp Outcome Assessment, Health Care/

14. exp Health Knowledge, Attitudes, Practice/ or exp "Surveys and Questionnaires"/ or exp Attitude to Health/

15. exp Patient Reported Outcome Measures/

16. exp Formative Feedback/ or exp Feedback/

17. exp Primary Health Care/ or exp "Delivery of Health Care"/

18. exp Dental Care/

19. exp Pharmacy Service, Hospital/ or exp Community Pharmacy Services/

20. exp Elective Surgical Procedures/

21. exp Hospitals/

22. exp Health Services/

23. natural language processing.mp. or exp Natural Language Processing/ or NLP.mp. or sentiment analysis.mp. or exp Sentiment Analysis/ or Latent Dirichlet allocation or Named entity recognition or topic modelling or text classification or keyword extraction

24. 1 or 2 or 3 or 4 or 5

25. 6 or 7 or 8 or 9 or 10 or 11 or 12 or 13 or 14 or 15 or 16

26. 17 or 18 or 19 or 20 or 21 or 22

27. 23 and 24 and 25 and 26
