# Supplementary material for: The Use of Natural Language Processing to Interpret Unstructured Patient Feedback on Health Services: Scoping Review
Source: J Med Internet Res. 2025 Aug 14;27:e72853. doi: 10.2196/72853 (PMC12352804; doi:10.2196/72853)
Supplement: Multimedia Appendix 2 [file jmir-v27-e72853-s002.docx]

**Multimedia Appendix 2**: Data extraction tool

| **Ref no** | **Title** | **Authors** | **Publication year** | **Journal** | **Data source(s) of unstructured feedback** | **Health care context** | **Number of texts used** | **Number of healthcare professionals rated** | **Type of health professional** | **Type of rating** | **NLP methods employed** | **Sentiment Analysis** | | **Topic Modelling** | | **Text Classification** | | **Key findings** |
| --- | --- | --- | --- | --- | --- | --- | --- | --- | --- | --- | --- | --- | --- | --- | --- | --- | --- | --- |
|  |  |  |  |  |  |  |  |  |  |  |  | Model used | Performance metrics | Model Used | Performance metrics | Model Used | Performance metrics |  |
| **1** |  |  |  |  |  |  |  |  |  |  |  |  |  |  |  |  |  |  |
| **2** |  |  |  |  |  |  |  |  |  |  |  |  |  |  |  |  |  |  |
| **3** |  |  |  |  |  |  |  |  |  |  |  |  |  |  |  |  |  |  |
| **4** |  |  |  |  |  |  |  |  |  |  |  |  |  |  |  |  |  |  |
| **5** |  |  |  |  |  |  |  |  |  |  |  |  |  |  |  |  |  |  |
| **6** |  |  |  |  |  |  |  |  |  |  |  |  |  |  |  |  |  |  |
| **7** |  |  |  |  |  |  |  |  |  |  |  |  |  |  |  |  |  |  |
| **8** |  |  |  |  |  |  |  |  |  |  |  |  |  |  |  |  |  |  |
