# Supplementary material for: The Use of Natural Language Processing to Interpret Unstructured Patient Feedback on Health Services: Scoping Review
Source: J Med Internet Res. 2025 Aug 14;27:e72853. doi: 10.2196/72853 (PMC12352804; doi:10.2196/72853)
Supplement: Multimedia Appendix 3 [file jmir-v27-e72853-s003.docx]

**Multimedia Appendix 3: Summary tables**

Summary table1: Sentiment analysis papers

| Study | Study setting | Aim of study | Method used | Summary of findings |
| --- | --- | --- | --- | --- |
| Yazdani et al (2023) [61] | - Collected 3600 feedback forms between March and October 2021 from the hospital website focusing on cancer patients. | - Detect cancer patients' sentiments on healthcare services in Persian language.  - Combine sentiment analysis and topic modelling for healthcare service insights. | Sentistrength  Topic modelling | - SA model achieved accuracies of 89.3%, 92.6%, and 90.8% in detecting sentiments.  - Patients expressed dissatisfaction with appointment booking service.  - System quality and interaction quality received majority of positive feedback. |
| Vasan et al. (2023) [33] | - Analysed 18546 reviews of 1240 otolaryngologists from the Healthgrades.com in March 2022. | - Analyse online reviews to uncover patterns among otolaryngologists' ratings.  - Determine factors influencing sentiment and star scores in physician reviews. | VADER | - Younger and male otolaryngologists had higher sentiment and star scores.  - Positive reviews associated with words like 'confident', 'kind', 'recommend', 'comfortable'.  - Negative reviews had words like 'pain', 'rude', 'problems', 'wait'. |
| Tang et al. (2023) [34] | - Analysed 7638 reviews of 786 hand surgeons from healthgrades.com and webmd.com. | - Quantitatively analyse hand surgeons' reviews using sentiment analysis.  - Report unbiased trends in words used to describe reviewed surgeons. | VADER | - Positive reviews linked to younger surgeons, good bedside manner.  - No significant difference in review sentiment by provider sex. |
| Tang et al. (2022) [35] | - Collected 5121 reviews of 721 spine surgeons from healthgrades.com. | - Quantitatively analyse SRS surgeon reviews through sentiment analysis.  - Determine relationship between demographic variables and average sentiment score. | VADER | - Sentiment scores correlate with star ratings, positive reviews for younger surgeons.  - Positive reviews linked to compassionate care, efficient pain management.  - Negative reviews associated with inability to relieve pain. |
| Tang et al. (2023) [36] | - Collected 8357 reviews of 480 spine surgeons from healthgrades.com. | - Analyse online reviews of spine surgeons for biases and trends.  - Assess sentiment analysis impact on patient provider selection. | VADER | - Younger male surgeons rated higher.  - Pain management crucial for positive reviews.  - Positive reviews linked to personal characteristics. |
| Tang et al. (2023) [37] | - Collected 2239 reviews of 177 spine surgeons from healthgrades.com. | - Quantitatively analyse CSRS surgeons' online reviews for biases and trends.  - Evaluate sentiment scores and word frequency in physician reviews. | VADER | - Positive reviews linked to younger surgeons and behavioural attributes.  - Pain management crucial for online reputation of CSRS surgeons.  - Office environment impacts online reviews favourably. |
| Shah et al. (2021) [52] | - Collected 55612 reviews of 3430 doctors from ratemds, healthgrades.com, and vitals. | - Identify sentiment trends in physician rating websites.  - Provide insights into patient opinions during the COVID-19 crisis. | Crystalfeel | - Negative emotions prevalent, fear, anger, and sadness during COVID-19.  - Emerging topics focused on treatment process, awareness, and COVID-19 control.  - Fading themes included panic buying, daily life impact, and bedside manner. |
| Shah et al. (2021) [53] | - Collected 152729 reviews of 10232 doctors from ratemds, healthgrades.com, and vitals. | - Investigate top concerns and sentiment dynamics in physician online reviews. | Crystalfeel  SenticNET | - Sentiments shifted from positive to negative to positive during COVID-19.  - ABSA captured sentiments towards healthcare quality aspects in online reviews. |
| Sewalk et al. (2018) [38] | - Collected 2759257 tweets related to healthcare experience between February 2013 and February 2017. | - Characterize patient experience sentiments on Twitter across the United States.  - Examine differences in patient views of health care spatially and temporally. | VADER | - Metropolitan areas show higher positive tweets compared to non-metropolitan areas.  - Decrease in tweet volume, increase in sentiment observed during study period. |
| Serrano-Guerrero et al. (2024) [21] | - Collected over 200,000 reviews from CareOpinion about healthcare professionals, covering a span of 5 years. | - Compare patient opinions on nurses and doctors for service improvement.  - Assess patient needs and satisfaction to enhance healthcare services. | VADER | - Patients' opinions on nurses more positive than doctors. |
| Quinones et al. (2022) [39] | - Collected 6815 reviews of 1284 neurosurgeons in the USA from healthgrades.com in the period of July to August 2020. | - Investigate patient-physician interactions and key phrases in neurosurgeon reviews. | VADER | - No difference in sentiment scores based on sex of neurosurgeons.  - Male neurosurgeons scored higher star ratings than females. |
| Park et al. (2023) [40] | - Collected 6400 reviews of 400 psychiatrists in the USA from healthgrades.com. | - Analyse patient reviews of psychiatrists on physician rating websites.  - Identify factors influencing patient perception of psychiatrists.  - Explore relationships between demographic factors, star ratings, and sentiment scores. | VADER | - Sentiment scores correlated with average star ratings of psychiatrists significantly.  - Positive reviews mentioned "time" and "caring," negative reviews mentioned "medication."  - Younger psychiatrists in the Northeast received higher star ratings. |
| Nawab et al. (2020) [16] | - Collected 2830 reviews from Press Ganey Associates about a hospital in the USA. | - Utilize NLP to mine free-text data for valuable information. | Neural networks | - Doctors and nurses received most comments, room had negatives.  - Negative comments on room, tests, and discharge categories.  - Climate control, housekeeping, and noise levels major concerns in rooms. |
| Menendez et al. (2019) [57] | - Collected 254 reviews from Press Ganey Associates on an orthopaedic hospital in the USA. | - Analyse factors linked to negative comments in patient experiences post-surgery. | A 3^rd^ party tool (Press Ganey Associates’ NLP tool) | - Patient-narrative analysis highlights care aspects critiqued by patients.  - Negative comments themes include room condition, communication, and pain management.  - Study population characteristics show age, sex, and insurance status distribution. |
| Li et al. (2022) [54] | - Collected 58600 reviews about doctors in China from haodf.com | - Investigate changes in patient attitudes towards doctors during COVID-19 pandemic.  - Analyse factors influencing improvements in doctor-patient relationships post-pandemic. | A 3^rd^ party tool (Baidu Cloud) | - Improved doctor-patient relationships post-COVID-19 in China.  - Decrease in negative reviews, shift towards positive attitudes post-pandemic.  - Sentiment analysis showed slight increase in positive comments during the pandemic. |
| Levy et al. (2023) [41] | - Collected 6606 reviews from healthgrades.com about 1854 urologists in the USA. | - Identify factors affecting online reviews for urologists nationwide. | VADER | - Older age doctors linked to more negative reviews in urologists.  - Positive reviews tied to physician's attitude and character phrases.  - Negative reviews associated with long wait times and patient pain. |
| Langerhuizen et al. (2021) [56] | - Collected 11614 reviews of orthopaedic surgeons from Yelp in the USA. | - Identify themes in online reviews of orthopaedic surgeons.  - Analyse patient experience based on review ratings and tones. | 3^rd^ party (IBM Watson) | - Themes varied by rating category, including logistics, care, trust, and recommendation.  - Positive reviews were shorter, with joy and confidence tones.  - Negative reviews had sadness and tentative tones. |
| Khanbhai et al. (2022) [43] | - A total of 69285 reviews were collected at a large London NHS Trust hospital. | - Extract themes and sentiment from patient experience comments using NLP.  - Identify issues in transitions of care across different healthcare settings. | SVM | - Negative sentiments include 'discharge', 'appointment', and 'home'.  - Tri-grams highlight issues like 'seeing different doctor' and 'information aftercare lacking'.  - NLP identifies problems with care transitions and continuity effectively. |
| Khaleghparast et al. (2023) [49] | - A total of 822 reviews were collected from a cardiovascular hospital in Iran. | - Perform sentiment analysis on patients' messages using lexicon-based and ML methods.  - Identify positive and negative comments and determine hospital ward and staff names. | - AdaBoost  - Decision Tree  - Logistic Regression  - Multilayer Perceptron  - Naïve Bayes | - Best classifier: Multinomial Naïve Bayes with MF + TFIDF feature vector.  - Previous studies used SA and ML to classify patients' comments effectively |
| Kao et al. (2015) [50] | - Collected 63 reviews of an academic multidisciplinary pain centre. | - Develop machine-mediated classification of patient feedback using NLP.  - Optimize patient experience monitoring with low cost and resource burden. | Recurrent Neural Networks (RNN) | - The optimal cut-off for classifying the feedback as Negative is a threshold of 68.5% or more of the sentences being Negative.  - There is moderate agreement between machine-mediated rating with single-rater coding, with Kappa of 0.57 and AUC of 0.79 |
| Jung et al. (2015) [76] | - Collected 173748 reviews of a paediatric hospital in South Korea from online communities | - Identify hospital service quality factors and trends from online health communities. | Keyword-based approach | - Service quality factor detection and hospital name extraction achieved average F1 scores of 91% and 78%, respectively |
| Jiménez-Zafra et al. (2019) [44] | - A total of 743 reviews of doctors with 34 specialties were collected from Masquemedicos, a medical community in Spain. They also collected reviews on drugs. | - Examine sentiment expression in medical forums for sentiment analysis methods.  - Analyse language to determine best approach for sentiment analysis. | - SVM  - iSOL Lexicon | - Drug reviews challenging, informal language with specific terminology.  - Physicians' reviews informal, easier to classify than drug reviews.  - Reviews about drugs longer, opinions about physicians have more adjectives. |
| Huppertz and Otto (2018) [45] | - Collected 57985 reviews from 113 hospitals’ Facebook page in the USA. | - Social media feedback predicts hospital HCAHPS scores accurately.  - Sentiment analysis on Facebook comments enhances patient experience assessment. | - Naïve Bayes  - KNN  - SVM | - Both number of stars and the number of positive comments posted on hospitals Facebook Reviews sections were associated with higher overall ratings and willingness to recommend the hospital.  - The findings suggest that patients’ informal comments help predict a hospital’s formal measures of patient experience. |
| Hu et al. (2019) [55] | - Collected approximately 29 million records from WeChat and Qzone in China about healthcare services. | - Identify content volume and sentiment polarity of healthcare services in China.  - Analyse public perceptions on healthcare services using social media data. | 3^rd^ party (Tencent NLP platform) | - Patient safety most discussed topic, negative sentiments in doctor-patient relationship.  - Sentiment analyses showed 36.1%, 16.4%, and 47.4% of positive, neutral, and negative emotions, respectively.  - Neutral disposition was found to be the highest (30.4%) in the contents on appointment-booking services. |
| Hawkins et al. (2016) [48] | - Collected 404065 tweets about hospitals in the USA | - Assess Twitter as a measure of patient-perceived quality in hospitals.  - Compare patient sentiments on Twitter with established quality measures. | - Naïve Bayes  - SVM | - Positive sentiment on Twitter, hospitals interact with users for feedback.  - Hospitals with more tweets had higher sentiment, no link to readmission. |
| Gui and He (2021) [51] | - Collected 201246 reviews on Yelp about healthcare services | - Propose joint learning framework for aspect extraction and sentiment classification | - Convolution Neural Network (CNN)  - Recurrent Neural Network (RNN)  - Long-Short Terms Memory (LSTM) | - Aspect-based model outperforms existing neural models for sentiment classification.  - Aspect-based model extracts coherent aspect topics and learns good document representations.  - Aspect-based model visualisation shows word and sentence-level attention weights in aspect-based. |
| Gour and Kumari (2021) [58] | - Collected 9492 reviews on hospitals in India from Various websites such as Facebook, Quora, MouthShut, Google reviews and Just Dial. | - Develop fuzzy sentiment analysis model for hospital patient reviews.  - Analyse reviews to help hospitals find pros and cons. | Naïve Bayes | - Traditional vs. fuzzy sentiment analysis showed significant differences in review sentiments.  - Trust and positive emotions were prominent in the hospital reviews.  - Negative emotions were more prevalent in fuzzy sentiment analysis results. |
| Pandey et al. (2023) [42] | - Collected 178 reviews of 34 General Practitioners (GPs) in Northamptonshire, UK from NHS website. | - Improve patient experience in healthcare.  - Classify GPs based on feedback.  - Enhance patient feedback processes. | VADER | - Classifier grouped GPs into gold, silver, and bronze categories.  - 10-fold cross-validation test mode for data set classification. |
| Almorox et al. (2022) [80] | - Collected 2195 reviews of 242 care homes in the UK from carehome.co.uk. | - Examine sentiment changes in care home reviews during COVID-19.  - Assess impact of integrated care on residents' and carers' experiences. | AFINN | - Care home sentiment increased during COVID-19, especially with integration terms.  - Staff role crucial in driving sentiment for residents and relatives. |
| Alemi et al. (2012) [46] | - Collected 995 reviews of 200 Paediatricians and obstetricians/gynaecologists in the USA from ratemds.com | - To demonstrate sentiment analysis feasibility in real-time satisfaction surveys.  - To classify patients' reasons for dissatisfaction using sentiment analysis. | - Decision trees  - bagging with decision trees  - Naïve Bayes  - SVM | - Sentiment analysis classifies comments into complaints or praise accurately.  - Majority of patient reviews and comments are positive and constructive. |
| Agarwal et al. (2022) [47] | - Collected approximately 10000 tweets in India about healthcare services. | - Extracting real-time Twitter data concerning three healthcare sub-domains: COVID-19 vaccine, post-COVID-19 health factors, and healthcare service providers.  - Using machine learning techniques to classify and analyse the sentiments and narratives expressed by social media users regarding these sub-domains. | - Support Vector Machine (SVM)  - Logistic Regression  - Random Forest  - Multinomial Naive Bayes (MNB)  -Long Short-Term Memory (LSTM) | - Sentiment analysis of the collected tweets provided insights into public opinion regarding the effectiveness of COVID-19 vaccines, long-term health impacts post-COVID, and the performance of healthcare service providers.  - SVM model showed highest accuracy of 82.67% compared to others. |

Summary table2: Topic modelling papers

| Study | Study setting | Aim of study | Method used | Summary of findings |
| --- | --- | --- | --- | --- |
| Yazdani et al (2023) [61] | - Collected 3600 feedback forms between March and October 2021 from the hospital website focusing on cancer patients. | - Detect cancer patients' sentiments on healthcare services in Persian language.  - Combine sentiment analysis and topic modelling for healthcare service insights. | LDA | - The topic "Metastasis" exhibited lower sentiment scores compared to other topics.  - Cancer patients expressed dissatisfaction with the current appointment booking service.  - Topics such as "Good experience," "Affable staff", and "Chemotherapy" garnered higher sentiment scores. |
| Stokes et al. (2021) [62] | - Collected 8133 Yelp reviews of 1383 mental health treatment facilities. | - Evaluate correlation between review themes, facility characteristics, and ratings. | LDA | - Positive themes: caring staff, non-pharmacologic treatment.  - Negative themes: rude staff, safety and abuse.  - Patient satisfaction linked to staff-patient communication.  - Reviews highlight concerns on safety, communication, and staff attitudes. |
| Ranard et al. (2016) [7] | - Collected 16862 Yelp reviews of 1352 hospitals. | - Compare Yelp reviews with Hospital Consumer Assessment of Healthcare Providers and Systems (HCAHPS) survey domains.  - Identify topics correlating with Yelp ratings and HCAHPS overall ratings. | LDA via MALLET | - Yelp ratings correlate with HCAHPS overall ratings.  - Yelp reviews cover more topics than HCAHPS survey.  - Yelp topics reveal major drivers of patient experiences. |
| Lin et al. (2020) [27] | - Collected online reviews of 204,751 dentists from HealthGrades.com | - Characterize online reviews of dental patients in the United States.  - Examine association between dentist characteristics and patient ratings. | A semi-automated NLP method to perform text mining (bigram and trigram), focusing on identifying relevant concepts. Then manually assessing the collocations manually by 2 raters. | - Higher ratings were associated with female dentists.  - Patient ratings linked to dentist characteristics and patient experience topics.  - Topics that corresponded to CAHPS measures, including discomfort (painful/painless root canal or deep cleaning), and ethics (high-pressure sales, and unnecessary dental work) |
| Lester and Chui (2017) [65] | - A total of 1,421 user reviews from 423 unique pharmacies were collected from Yelp. | - Identify patient topics at community pharmacies and their impact on Yelp ratings.  - Improve patient experience and provide actionable feedback for pharmacies. | LDA | - Topics from Yelp reviews inform pharmacy improvement for patient experience.  - Identified four discernible topics including long wait times for prescriptions, helpful pharmacy staff, good store environment, and issues filling medications. |
| Graves et al. (2018) [81] | - Collected 42752 Yelp reviews on hospitals in the US regarding the pain management and opioids. | - Characterize Yelp reviews on pain management and opioid experiences.  - Identify themes impacting satisfaction and patient experiences with opioids. | Differential language analysis (DLA) | - Patients describe negative factors like long wait times and poor pain control.  - Positive aspects include attentive medical staff.  - Reviews show perceptions of excessive opioid use and inappropriate care. |
| Chekijian et al. (2021) [82] | - Collected 6406 anonymised reviews from Press Ganey about hospitals. | - Understand patient experience during COVID-19 using sentiment analysis and topic modelling.  - Identify key trends in sentiment and topics related to patient experience. | LDA | - Patient concerns shifted pre, during, and post-COVID-19 with varying sentiments.  - Topics included safety, family involvement, and system issues during COVID-19.  - Positive sentiments decreased during COVID-19 but increased post-COVID-19. |
| Chan et al. (2022) [83] | - Collected 619 Yelp reviews on crisis pregnancy centres in California. | - Analyse patient experiences and motivations at California Crisis Pregnancy Centres (CPCs).  - Evaluate crowdsourced reviews of CPCs using Yelp. | Thematic coding validated by meaning extraction method. | - Motivations for CPC care: pregnancy confirmation, emotional support, abortion care.  - CPC experiences: mixed, faith-based practices, inaccurate medical information, lack of transparency.  - Patients seek CPCs due to gaps in healthcare coverage, free materials.  - CPCs serve socially vulnerable groups, including uninsured and low-income patients. |
| Cammel et al. (2020) [74] | - Collected 38664 patients’ experience data from 2 hospitals. | - Categorize patient responses using unsupervised topic modelling.  - Create combined sentiment and frequency measure for improvement prioritization.  - Assess model transferability on free-text responses from another hospital. | - Non-negative matrix factorization (NMF)  - Latent Dirichlet Allocation (LDA) | - the first hospital resulted in 127 topics and 294 n-grams.  - The indicator ‘impact’ revealed n-grams to celebrate (15.3%), improve (8.8%), and monitor (16.7%).  - Between-hospitals, most topics (69.7%) were similar, but 32.2% of topics for hospital 1 and 29.0% of topics for hospital 2 were unique. |
| Agarwal et al. (2019) [59] | - Collected 100949 Yelp reviews on urgent care centres. | - Compare online reviews of EDs and urgent care centres.  - Identify topics correlated with 1-and 5-star ratings in reviews. | LDA | - Patients value comfort, professionalism, facilities, and staff interactions.  - Themes associated with 5-star reviews among EDs and urgent care centres were similar for comfort, professionalism, facilities, paediatric care, and staff interactions.  - Themes associated with 1-star reviews among EDs and urgent care centres were similar for communication, telephone experience, waiting, billing, pain management, and diagnostic testing.  - Themes unique to 5-star ED reviews included bedside manner, care for family members, and access. Themes unique to 5-star urgent care centre reviews were based on recommendation and prescription refills. Themes unique to 1-star ED reviews were service and speed of care. Themes unique to 1-star urgent care centre reviews were lack of confidence and reception experience. |

Summary table 3: Text classification papers

| Study | Study setting | Aim of study | Method used | Summary of findings |
| --- | --- | --- | --- | --- |
| Rajagopalan et al. (2022) [84] | - Collected 1390 reviews on various specialties of doctors in the USA | - Develop quantitative patient experience measures using natural language processing.  - Address ceiling effects in patient-reported experience measures. | - Linguistic Inquiry and Word Count (LIWC)  - Thematic analysis | - Analysis identified 36 emotional and linguistic constructs associated with  ordinal rating of likelihood to recommend  - LIWC used to summarize text comments for likelihood to recommend ratings.  - the score developed from thematic analysis both had a relatively  normal distribution and limited or no ceiling effect. |
| Parikh et al. (2024) [73] | - Collected 2292 reviews about radiology of a hospital department in the USA. | - Describe effects of demographics and examination factors on MRI experience.  - Understand patient feedback for patient-centred changes. | - Sentence BERT  - Multilabel XGBoost | - Male gender linked to positive ratings, shorter exams, and punctuality.  - Longer wait times and exams associated with negative patient ratings.  - Positive feedback themes: excellent service, on-time appointments, comfortable IV placement.  - Negative feedback themes: long wait times, poor communication, physical discomfort. |
| He et al. (2020) [72] | - Collected 1,065,631 reviews on 102,540 doctors in the USA from Vitals website. | - Identify patient concerns in online physician reviews through qualitative analysis.  - Improve healthcare providers' ratings based on consumer feedback. | - Manually labelled 600 reviews and then trained a classifier. | - Relationship is a key concern in patient reviews.  - Management issues trigger negative reviews more than clinical performance.  - Fine-grained aspects include timing, access, finance, and communication. |
